# Some Professional Reminiscences1Read before the Charaka Club, N. Y.

**Published:** 1907-04

**Authors:** George F. Shrady

**Affiliations:** New York


### SELECTED ARTICLE—SPECIAL.

### Some Professional Reminiscences.[1]

By GEORGE F. SHRADY, M. D., New York.

*(Journal American Medical Association, February 16, 1907.)*

EVERY one who travels even an ordinary road has experiences of his own. If he can apply them to the good of any of his fellow-creatures he is serving in a very general way the common interests of humanity. There is always something, if he only turns his head in the right direction, that he may see in a new light.

At certain times we react more sharply to the controlling circumstances of environment than ordinarily. It is the question of the man, the gun, the aim and the game being in proper line. Too often, alas! we not only miss the mark, but get kicked by the recoil. Thus the physician, by virtue of his judicial office, soon learns not only to think before he speaks, but oftentimes not to say what he thinks. His face must never be the mirror of his soul. To manifest surprise at any unexpected change in the illness of a patient is to confess ignorance. Even death itself must always be viewed in the light of any other accident. To the one seeking advice it is always a comfort to know that his physician has well grounded and positive opinions. In fact, he can the better trust him when he is sure that his faithful adviser is never wrong. If it were otherwise, there would be no need for doctors. Filled with such heretical thoughts, I am tempted to relate some personal experiences substantiating them.

After finishing my hospital interneship and imagining that I was well prepared for the practice of the noble art, I was summoned to a sick child in a tenement. The little one was lying in a cradle, playing quite unconcernedly with a rag doll. "Not very sick," I thought; "but what can be the matter with her?" I certainly had never seen the like symptoms before. There was a general spotted eruption over the face and other exposed places, a rasping cough, bleared, lachrymose eyes and a profuse coryza

---

1. Read before the Charaka Club, N. Y.

Not smallpox, I was sure, nor its reputed relation, as I had seen much of both.

"An ex-house surgeon of New York Hospital must not be worsted by such a case as this. There is one thing," said I to myself, "she is not going to die in any event." Just then the grandmother of the child came from a washtub in an adjoining corner of the room and seriously added to my embarrassment by squarely and rather disrespectfully, I thought, plumping out the question: "Docther, what is the mather with the choild?"

For self-protection I gathered my consciousness within myself, dropped my countenance curtain, and manifested a duly abstracted air, and unconcerned manner, while I took out my prescription book and pretended to be writing. I found it very convenient at that time not to hear her question. Still she stood there defiantly, with arms akimbo, with smell of suds and look of scorn, and repeated her patronizingly insistent demand. No washerwoman had ever dared do so before.

It was nevertheless trimming to a cross sea with set sails and a gathering storm. After all, she was only repeating my query. There was one other in that room that looked for the answer more eagerly than she could imagine. To gain time he took refuge in dazed and abstracted thought, adjusted his stethoscope and mutely motioned the clamorous interloper to be silent.

The bared chest showed more spots. The patient cried in fear, and due time was consumed in quieting her. The responsibility of the situation became more and more oppressive. The sonorous grunts of my critic overwhelmed any possibly abnormal auscultatory signs. The only urgent thing was the diagnosis. The last resource was in subterfuge and hypocrisy; anything but a humiliating acknowledgment of ignorance.

Again: "Docther, why don't you tell me what is the mather with the choild?"

"O yes," said I; "the fact is you would not understand it if I gave the name for the disease. The Latin term is *conjunctivitis,* and, of course, that would mean nothing to you."

Her supercilious and incredulous smile I shall never forget. Her rejoinder was electric. A new light dispelled the fog. "And is that the Latin name for *measles?*" said she.

I could have hugged the woman, suds and all, but my studied professional reserve came to my relief. "No, not the name for the *measles,* but I thought you wanted to know what ailed her eyes." Still the old woman unconsciously saved me from utter defeat and gave me my first clinical demonstration of the disease. Since then it has always been my religious duty to treat the grandmother in the sick room with becoming consideration.

Inasmuch as the patient is always the uncert[illegible]n factor in the equation of chances in making a prognosis, it is always safe to be on the qualifying side of possible antagonistic conditions. Thus we all learn never to be surprised at anything that may happen.

Many years ago I was treating a young lady for typhoid fever. While she was convalescing I had been absent from town for a day or two. On my return by an evening train I called to see her on my way home. It was a casual visit, and anything unusual was far from my expectation. On attempting to ring the door bell in a dimly-lighted street my hand grasped an ominous fold of crape. I looked at the number to be sure I was at the right place. "Dead! What could have happened during my absence? Possibly perforation of the bowel or internal hemorrhage. I have been away and some other man has been called in the emergency. In any event now is the time to say little and listen."

When admitted I went at once to the room, stood on the landing for a moment and heard various voices within. "They are laying out the corpse," thought I, "and my call will be a consolatory function."

In response to my knock the door was opened by the sister, who surprised me with a smiling welcome. I walked to the center of the room and saw my patient sitting by her bed!

"Oh! doctor, we are so glad you called. Josie has been longing for you for days. She wishes to ask," continued her sister, "if she can eat some oysters."

As for the astonished physician, no actual surprise must be shown. Oysters on ice instead of my patient!

"How many oysters will satisfy you?" was my question.

"Can I have six on the half shell?"

"Yes, order eight, with crackers and lemon."

Then, taking her hand in mine and patting it encouragingly, I found myself saying: "Josie, I am so glad, so glad——"

"Glad for what?" inquired she.

"Glad that you like oysters, as I was afraid that you might not be able to eat them tonight." Here truth hypocritically shook hands with policy.

When in the hall again the sister asked me if I had not been taken aback at finding crape on the bell, at the same time explaining that an old boarder had died in the room above. The dart had simply shied a little.

"Surprised? Why, no. I knew it could not have been Josie. Why should she die now when she is recovering?" Yet for the time being I was seemingly nearer to a corpse than I cared to be again. Of course, the patient never heard of the circumstance until long afterward, and luckily for her is still able to enjoy her oysters.

In my earlier journalistic experience I conceived the possibility of giving due variety to editorial functions by contributing an anonymous criticism on a paper that had been read before the Medical Society of the State of New York at Albany. The tone of the communication was as respectful and courteous as could be made by one who disputed the conclusions of the author, and asked for more light. The writer of the original article was, besides, a personal acquaintance who had an abundant ability to make a suitable reply.

As no answer came for a fortnight or more, I was somewhat chagrined by imagining that I had merely fired a blank cartridge thought on a half-rock calculation. This illusion, however, vanished in due time by the appearance of my opponent, who came specially from Albany to consult me on the propriety of his answering his critic. He was extremely anxious to know who had the temerity to differ from him, but in his excited state of mind I concluded that any direct statement to that effect should be a matter of subsequent and deliberate reflection. In any event, he wished me to help him make his arguments as striking as possible. There was, indeed, a ludicrously pathetic side of the situation that appealed to me, and I did my friendly duty by making several suggestions which he accepted with extreme satisfaction, and at the same time assuring him that he would effectually silence his presumptuous assailant.

The reply was duly published and I took occasion to congratulate him on his victory. It so happened that, his paper having received so much well-merited recognition, he was invited to elaborate it and present it to the Academy of Medicine. On that occasion a member who was always ready to discuss any subject rose and, to my great surprise, used all the points of argument contained in the anonymous attack. This was enough for the author of the paper. Turning to me with smiling satisfaction, he whispered: "At last I've found the fellow. Now watch while I wind him up."

The unconscious victim little imagined that he had short-circuited a live wire that had been charging itself for months. The result was startling and overwhelming. The Academy was spell-bound at the vehemence of the author's invective sarcasm, and when the excited speaker took his seat it was plain that the victory was entirely on his side. The only comfort for the vanquished one was complacently to blow and cool the finger that he had so foolishly put in the fire.

But this was not the end of the matter. For a long time afterward, whenever the volunteer critic appeared at the state meeting with a paper, he was sure to have one, at least, who would take issue with him. Nor did they ever after exchange views other than at safe and respectful distance in the debating arena. The explosion of mutual antagonism had blown them so far apart that each was on the opposite side of any possible reconciliation. Nobody, however, knew better than I that both were at fault for meddling with somebody else's fireworks; and so at one of the meetings I, with an innocence I could not feel, asked my Albany friend, "Why do you always worry Dr. G.?"

"Do you not remember his published criticism of my paper on fractures?" replied he.

"But he did not write it," was the rejoinder.

"Who then?"

"It was I."

"How absurd it was," he laughingly said, "that I should have come to you, of all others, for help under the circumstances."

"Yes! But didn't I do my best to help you silence the real man?" was the only excuse I could offer.

And so in my conscience-stricken way I satisfactorily untangled a ludicrous snarl of misdirected purposes.

